# Dietary Vitamin D_3_ Modulates Antioxidant Responses, Immune-Related Gene Expression, and Flesh Quality in Giant Freshwater Prawn (*Macrobrachium rosenbergii*) Under Pathogenic *Vibrio* Challenge

**DOI:** 10.3390/ani16162463

**Published:** 2026-08-07

**Authors:** Yan Liu, Qi Dong, Yuxuan Xu, Chencheng Hu, Chen Yang, Qun Jiang, Xiaojian Gao, Yao Zhang, Xiaojun Zhang

**Affiliations:** College of Animal Science and Technology, Yangzhou University, 48 Wenhui Road, Yangzhou 225009, China

**Keywords:** *Macrobrachium rosenbergii*, vitamin D_3_, non-O1 *Vibrio cholerae*, innate immunity, flesh quality

## Abstract

The giant freshwater prawn (*Macrobrachium rosenbergii*) is a commercially pivotal crustacean species in global freshwater aquaculture, while vibriosis caused by non-O1 *Vibrio cholerae* has become the primary bottleneck restricting its sustainable intensive farming. Vitamin D_3_ (VD_3_) is a biologically active fat-soluble nutrient involved in mineral metabolism and immune regulation in animals, yet its effects on the physiological responses of *M. rosenbergii* under a pathogenic bacterial challenge remain largely unelucidated. In this study, six experimental diets were formulated with graded dietary VD_3_ concentrations (0.10, 0.18, 0.28, 0.48, 0.68, and 0.88 mg/kg) to feed juvenile *M. rosenbergii* (initial body weight: 2.06 ± 0.07 g) for 70 d, followed by a 72 h non-O1 *Vibrio cholerae* challenge test. The results showed that dietary VD_3_ supplementation alleviated the non-O1 *Vibrio cholerae*-induced histopathological damage in the intestine and hepatopancreas of prawns. VD_3_ supplementation significantly modulated antioxidant enzyme activities in a tissue-specific manner: SOD activity increased in the intestine, while CAT and GSH-PX activities increased in the hepatopancreas. However, the MDA, T-AOC, and GSH-PX (intestine) or SOD (hepatopancreas) showed no significant responses, indicating that VD_3_’s antioxidant effects were selective rather than comprehensive. Meanwhile, an appropriate amount of VD_3_ markedly upregulated the mRNA expression of multiple immune-related genes (pattern recognition receptors, antimicrobial peptides and immune effectors) in a tissue-specific pattern. However, some antioxidant indices and immune-related genes showed no significant responses. Furthermore, VD_3_ supplementation significantly modulated certain muscle physicochemical parameters of prawns, including altered redness, cooking loss, and textural characteristics. Based on the evaluated physiological indicators, 0.48–0.68 mg/kg VD_3_ appeared to produce the most favorable overall response. These findings suggest that dietary VD_3_ may modulate the physiological responses associated with host defense during a bacterial challenge in *M. rosenbergii*, potentially through modulation of antioxidant capacity and immune-related gene expression, while benefiting the flesh quality. VD_3_ is thus a potential nutritional approach for improving health status during a bacterial challenge in freshwater crustacean farming.

## Simple Summary

Giant freshwater prawn is an important farmed species, but a bacterial disease called vibriosis causes major losses and antibiotic overuse raises safety concerns. This study investigated whether adding vitamin D_3_ to feed could enhance the physiological responses and flesh quality. Juvenile prawns were fed diets containing different vitamin D_3_ levels for ten weeks, followed by a bacterial challenge test. The results showed that the prawns fed moderate levels of vitamin D_3_ (0.48–0.68 mg/kg) had less tissue damage in the intestine and hepatopancreas, higher selected antioxidant enzyme activities, and changes in expression of immune-related genes. In addition, these prawns displayed altered muscle quality characteristics, including reduced cooking loss and changes in texture parameters. These findings indicate that appropriate dietary vitamin D_3_ supplementation can enhance the physiological responses associated with host defense during bacterial challenge and alter several texture parameters in farmed prawns, providing preliminary evidence for the application of vitamin D_3_ as a functional feed additive in freshwater prawn aquaculture.

## 1. Introduction

Global aquaculture is the fastest-growing food production sector worldwide, serving as a critical source of high-quality animal protein for the expanding global population [1]. The giant freshwater prawn (*Macrobrachium rosenbergii*) is a commercially pivotal species in global freshwater crustacean farming, favored for its rapid growth rate, premium flesh quality, and high market value [2]. In China, the annual production of *M. rosenbergii* has surpassed 196,374 tons, underpinning its role as a pillar industry for rural economic revitalization and aquaculture industrial upgrading [3]. However, the intensification of *M. rosenbergii* farming has led to a surge in bacterial disease outbreaks, most notably vibriosis [4], which has emerged as the primary bottleneck limiting its sustainable development.

Non-O1 *Vibrio cholerae*, a Gram-negative opportunistic pathogen ubiquitous in freshwater and brackish aquaculture systems, is a well-documented causative agent of mass mortality in farmed *M. rosenbergii* [4]. Infection with this pathogen induces severe systemic vibriosis in prawns, manifesting as hepatopancreatic necrosis, intestinal barrier disruption, and immune suppression [5]. Non-O1 *V. cholerae* produces extracellular virulence factors, including hemolysins and proteases, that disrupt epithelial barriers, and vibriosis outbreaks cause mass mortality and substantial economic losses in prawn farming [4,5,6]. Upon infection, prawns rely on innate immune defenses dominated by hemocyte-mediated phagocytosis, antimicrobial peptides, and the prophenoloxidase cascade [7,8]. The intestine and hepatopancreas are the primary target organs of *Vibrio* infection and the central sites of nutrient metabolism and immune regulation in crustaceans, and were therefore selected as the focal tissues in this study. Currently, antibiotic administration remains the dominant strategy for controlling aquaculture vibriosis, but the overuse of antibiotics causes product residues, ecosystem disruption, and public health risks [9]. Thus, safe and effective alternatives to antibiotics are urgently needed. Nutritional interventions using functional feed additives have been widely recognized as a sustainable strategy to improve disease resistance and growth performance in cultured crustaceans.

Vitamin D_3_ (VD_3_) is canonically recognized for its essential role in regulating calcium–phosphorus homeostasis and skeletal development in vertebrates [10]. Accumulating studies in mammals have uncovered extensive non-classical biological functions of VD_3_, including the regulation of antioxidant defense, innate immunity, anti-inflammatory responses, and cellular proliferation and differentiation [11,12,13]. In aquatic animals, dietary VD_3_ supplementation has been reported to improve growth performance, antioxidant capacity, and innate immunity in multiple teleost species and the Pacific white shrimp (*Litopenaeus vannamei*), the most-farmed penaeid shrimp worldwide [12,14,15,16]. Notably, these reported benefits of VD_3_ in crustaceans were obtained mainly under non-challenge conditions or environmental stress (e.g., low salinity) rather than under pathogenic infection [16,17,18]. Although crustaceans lack a vertebrate-type skeleton, they possess a complex calcium metabolism tightly coupled to the molting cycle, and dietary VD_3_ has been shown to affect the molting performance, tissue calcium–phosphorus deposition, and immune status in decapod crustaceans, such as the Chinese mitten crab [19]. To date, studies on VD_3_ in crustaceans have focused mainly on penaeid shrimp under non-challenge conditions or environmental stress, and the examined endpoints have largely been restricted to growth, antioxidant enzyme activities, and a limited set of immune parameters [16,17,18,20]. Which physiological responses respond to VD_3_ during actual pathogen infection, whether these responses are tissue-specific, and whether they occur in *M. rosenbergii* remain unanswered. Mechanistically, a canonical vitamin D receptor (VDR) homolog has not been functionally characterized in crustaceans, and it remains unclear whether the reported effects involve VDR-like genomic pathways, non-genomic mechanisms, or simply reflect an improved nutritional status. Nevertheless, the consistent responses of calcium–phosphorus deposition, molting performance, antioxidant capacity, and immune indices to dietary VD_3_ or 25-OH-D_3_ in decapod crustaceans [19,20] indicate that vitamin D metabolites are biologically active in this taxon, which justifies a systematic evaluation of VD_3_ under a pathogen challenge.

Accordingly, this study tested the hypothesis that dietary VD_3_ supplementation can attenuate non-O1 *V. cholerae* challenge-induced tissue damage and alter the muscle physicochemical properties in *M. rosenbergii* through enhancement of antioxidant defenses and modulation of innate immune responses. We formulated graded dietary VD_3_ concentrations to feed juvenile *M. rosenbergii* and conduct a non-O1 *Vibrio cholerae* challenge trial. We evaluated the histopathological changes, antioxidant enzyme activities, and immune-related gene expression in the intestine and hepatopancreas, alongside the muscle physicochemical properties across all treatment groups. To our knowledge, this study is the first to evaluate the effects of dietary VD_3_ on non-O1 *V. cholerae*-induced tissue injury in *M. rosenbergii* and its tissue-specific associations with antioxidant defense and innate immune gene expression. The muscle physicochemical properties were included as an integral endpoint because a bacterial challenge induces oxidative stress and tissue damage that can promote protein oxidation and impair the water-holding capacity and texture, whereas VD_3_-mediated regulation of calcium homeostasis and antioxidant status may counteract these processes [21]; the flesh quality is also a key economic trait of farmed prawns [22]. The selected immune-related genes cover the key functional layers of the crustacean innate immune system, namely pattern recognition, antimicrobial defense, the prophenoloxidase system, and immune regulation [7,8]. To our knowledge, limited information is available regarding the effects of dietary VD_3_ supplementation on the immune and antioxidant responses of *M. rosenbergii* during a *Vibrio* challenge, particularly its tissue-specific regulation of antioxidant defense and innate immunity. Our findings will provide a sustainable nutritional strategy for vibriosis control and quality improvement in freshwater prawn farming.

## 2. Materials and Methods

### 2.1. Experimental Diet Composition and Preparation

Supplementary Table S1 provides detailed information about the diets employed in this research. The basal diet was formulated to meet the nutritional requirements of *M. rosenbergii* and contained approximately 40.05% crude protein, 9.26% crude lipids, 10.30% ash, and 18.08 gross energy. The primary protein components incorporated into the diets included fish meal, soybean meal, wheat, soybean protein concentrate, and shrimp powder, while linseed oil and soybean oil served as the lipid sources. The experimental diets were formulated with graded supplementation levels of VD_3_ crystalline powder (purity ≥ 99%, Sigma-Aldrich, St. Louis, MO, USA) at 0, 0.10, 0.20, 0.40, 0.60, and 0.80 mg/kg above the basal diet. Corn starch was added to standardize the total weight of each diet. The actual measured VD_3_ concentrations in the final diets were determined by high-performance liquid chromatography with UV detection at 265 nm [23]. Briefly, the samples were saponified with ethanolic potassium hydroxide solution, extracted with n-hexane, purified through a silica solid-phase extraction cartridge, and separated on a C18 reverse-phase column (250 × 4.6 mm, 5 μm) with methanol:water (95:5, *v*/*v*) as the mobile phase at a flow rate of 1.0 mL/min. The final dietary VD_3_ concentrations were 0.10 (basal), 0.18, 0.28, 0.48, 0.68, and 0.88 mg/kg. The 0.10 mg/kg in the control diet represents the basal VD_3_ content derived from the natural ingredients (primarily fish meal and shrimp powder). The feed was prepared fresh every 4 weeks to minimize potential degradation in order to maintain VD_3_ stability. The dry matters were first mixed thoroughly and passed through an 80-mesh sieve, after which linseed oil and soybean oil were incorporated. Distilled water (at a 25% proportion) was then added to the mixture, which was subsequently processed into pellets with a 1.5 mm diameter using a twin-screw extruder (F-26, Industrial General Factory of South China University of Technology, Guangzhou, China). The prepared diets were air-dried and preserved at −20 °C until use.

### 2.2. Animal Adaptation and Rearing Environment

This experiment was carried out at the aquaculture facility of Jiangsu Freshwater Fisheries Research Institute, located in Yangzhong, Jiangsu Province. Juvenile prawns, sourced from a local aquaculture farm in Gaoyou, Jiangsu Province, were acclimated to the experimental environment for a two-week period. The control diet was fed to prawns during the domestication period. Only healthy juvenile prawns of uniform body size and in the intermolt stage were selected for the trial. No mortality occurred during the acclimation period, and all prawns showed normal behavior before grouping.

For the growth trial, 1440 juvenile prawns (mean initial weight: 2.06 ± 0.07 g) were randomly distributed into 18 outdoor concrete ponds (1.80 × 1.80 × 1.20 m) at a stocking density of 80 individuals each pond. The 18 experimental ponds were arranged in a completely randomized design. Each dietary treatment was randomly assigned to 3 ponds using a random number table. The ponds were randomly distributed within the experimental facility to minimize the positional effects. Each experimental group was replicated 3 times, with continuous aeration provided to all ponds throughout the 70-day period. The prawns were fed at 3–5% of body weight per day, adjusted every two weeks based on sample weighing (30 prawns per treatment). The daily ration was divided into three equal meals (07:00, 13:00, and 18:00). Uneaten feed was collected from the feeding trays after each feeding, dried at 60 °C and weighed. The actual feed intake was calculated in combination with the feed dissolution loss rate. Sand-filtered river water was used for the aquaculture and supplied to each pond. The culture ponds at the experimental base adopted a micro-flow water system, and approximately 30% of the pond water was exchanged every 3 days with sand-filtered river water. The water quality parameters were monitored daily and maintained within optimal thresholds: the dissolved oxygen was maintained above 8 mg/L, the water temperature ranged from 28 to 32 °C, and the pH was stabilized at 7.5 ± 0.3. Additionally, the ammonia-nitrogen and nitrite-nitrogen concentrations were strictly regulated below 0.1 mg/L and 0.03 mg/L, respectively. The growth performance data from this feeding trial were previously reported by Zhang et al. [23]; the present study focuses on the physiological, molecular, and flesh quality responses.

### 2.3. Bacterial Strain and Challenge Trial

The strain used in this experiment was isolated from the hepatopancreas tissue of naturally infected *M. rosenbergii* and preserved in our laboratory, which was identified as *Vibrio cholerae* non-O1 serogroup via 16S rRNA sequencing [6]. Prior to the experiment, a single colony was inoculated into LB liquid medium and cultured at 28 °C with shaking at 180 r/min for 16 h. The bacterial culture was then centrifuged at 5000 r/min (centrifugal radius: 5.4 cm) for 10 min, and the supernatant was discarded. The bacterial pellets were resuspended by pipetting with sterile phosphate-buffered saline (pH 7.2) and adjusted to a final concentration of 1 × 10^5^ cfu/mL. The bacterial suspension concentration was confirmed by serial 10-fold dilution and plating on thiosulfate–citrate–bile salts–sucrose agar, incubated at 28 °C for 24 h. Colony counts were performed in triplicate. Thirty *M. rosenbergii* individuals were randomly selected from each group of the growth trial and designated as the infected groups, with 3 replicate sets for each group. Each replicate contained 10 prawns. During the challenge trial, the prawns were maintained in 100 L polyethylene tanks (60 L water volume) with continuous aeration. The water temperature (28 ± 1 °C), dissolved oxygen (>6 mg/L), and pH (7.5 ± 0.3) were monitored daily. Based on previous tests, a bacterial suspension at a sublethal challenge concentration (1 × 10^5^ cfu/mL) was prepared [23]. Each individual in the infected groups was injected with 100 μL of the bacterial suspension using the intraperitoneal injection method. Each prawn received an injection dose of 1 × 10^4^ CFU (100 μL of 1 × 10^5^ CFU/mL suspension). At 72 h post-infection, some *M. rosenbergii* individuals exhibited disease symptoms, such as body reddening and reduced feeding activity. A sublethal challenge dose was selected based on the pre-experimental results, which induced significant infection symptoms and tissue injury without causing mortality within 72 h. This model was chosen to enable the systematic analysis of host’s early physiological and immune responses, as well as to ensure sufficient surviving individuals for tissue sampling across all groups.

### 2.4. Sample Collection

After the challenge trial, twenty-four individuals from each group were anesthetized with MS-222 (Millipore Sigma, St. Louis, MO, USA) for subsequent dissection of intestine and hepatopancreas tissues [23]. For the biochemical assays and qPCR analysis, 6 prawns per tank (18 per treatment) were anesthetized with MS-222, and the intestine and hepatopancreas were rapidly dissected, snap-frozen in liquid nitrogen, and stored at −80 °C. The tissues from individual prawns were analyzed separately (not pooled). For the histopathological analysis (H&E staining), 2 prawns per tank (6 per treatment) were fixed in 4% paraformaldehyde. Because the dietary treatments were assigned at the pond level, the pond was considered the experimental unit for the statistical analyses. The individual measurements within each pond were averaged before analysis.

### 2.5. Biochemical Assays Analysis

For the enzyme activity determination, the intestine and hepatopancreas tissues were ground into a powder in liquid nitrogen [23]. An appropriate amount of the ground tissue was transferred into a 1.5 mL centrifuge tube. The intestine and hepatopancreas tissues were homogenized in ice-cold 0.9% sterile saline at a ratio of 1:9 (*w*/*v*; 1 g tissue in 9 mL saline) to prepare a 10% (*w*/*v*) tissue homogenate. After incubation on ice for 30 min, the homogenate was centrifuged at 6000× *g* at 4 °C for 20 min, and the supernatant was collected for subsequent use. A suite of biochemical parameters, encompassing tissue protein concentration, malondialdehyde (MDA), catalase (CAT) activity, glutathione peroxidase (GSH-PX), superoxide dismutase (SOD), and total antioxidant capacity (T-AOC) were quantified using commercial assay kits purchased from Nanjing Jiancheng Bioengineering Institute (Nanjing, China). The MDA content, a biomarker of lipid peroxidation, was measured using the thiobarbituric acid method and expressed as nmol/mg protein. All enzyme activities and MDA contents were normalized to the protein concentration of the same tissue sample and expressed per milligram of protein. The analysis of biomarker and enzymes activity-related parameters is shown in Supplementary Table S2.

### 2.6. qPCR

The process of qPCR was based on the previous established method [23,24,25]. The total RNA was isolated from the tissues using TRIzol reagent (Aidlab Biotechnologies, Beijing, China). The purity and concentration of the extracted RNA were evaluated through agarose gel electrophoresis and spectrophotometric analysis (assessed by A260/280 nm absorbance ratio), respectively. The RNA integrity was further assessed by 1.5% agarose gel electrophoresis to visualize the 28S and 18S ribosomal RNA bands, and the RNA integrity number (RIN) was determined using an Agilent 2100 Bioanalyzer (Agilent Technologies, Santa Clara, CA, USA), with RIN > 7.0 for all samples. The total RNA was treated with RQ1 RNase-Free DNase I (Promega, Madison, WI, USA) according to the manufacturer’s protocol to eliminate genomic DNA contamination. Complementary DNA was synthesized utilizing a reverse transcription kit (Aidlab Biotechnologies, Beijing, China). First-strand cDNA was synthesized from 1 μg of total RNA with oligo(dT)18 primers in a 20 μL reaction volume. The reaction system for the qPCR consisted of 2 μL of cDNA template, 10 μL of green qPCR supermix, 0.8 μL of specific primers, and 7.2 μL of nuclease-free water (Aidlab Biotechnologies, Beijing, China). All the qPCR reactions were conducted on a QuantStudio™ 3 Real-Time PCR System (Thermo Fisher, Waltham, MA, USA) following the instrument manufacturer’s standard protocols. The thermal profile was set as follows: initial denaturation at 95 °C for 3 min, followed by 40 cycles of 95 °C for 10 s (denaturation), 58 °C for 20 s (annealing), and 72 °C for 20 s (extension). The primer sequences are listed in Supplementary Table S3. Primer specificity was confirmed by a melting curve analysis (single peak for all primers) and agarose gel electrophoresis (single band of expected size). No amplification was observed in any control reaction. All samples were run in technical triplicates, and the mean Cq value was used for quantification. The relative expression levels of target genes were calculated using the 2^−ΔΔCT^ method, with EF-1α serving as the internal reference gene. The stability of EF-1α as a reference gene was validated using geNorm and NormFinder algorithms, which confirmed its suitability as a stable reference gene under our experimental conditions (M < 0.5; stability value < 0.15).

### 2.7. Preparation of Paraffin Sections and H&E Staining

Samples fixed in 4% paraformaldehyde were first rinsed and trimmed, then vertically positioned in labeled plastic embedding cassettes [23]. These cassettes were subjected to gradient ethanol dehydration sequentially, including 75% ethanol for 2 h, 85% ethanol for 2 h, 95% ethanol for 3 h, and absolute ethanol for 1 h, followed by immersion in toluene for 20 min for clearing. After infiltration with a toluene–paraffin mixture at 58 °C for 2 h, the samples were embedded in molten paraffin using a HistoCore Arcadia embedding system (Leica Biosystems, Nussloch, Germany). Serial 5 μm thick sections were prepared with a HistoCore Multicut microtome (Leica Biosystems, Nussloch, Germany). Subsequent to H&E staining, the sections were observed and imaged under an Olympus BX43 light microscope (Olympus, Tokyo, Japan).

### 2.8. Shear Force, Flesh Texture, Cooking Loss, and Color Analysis

The muscle shear force, texture profile, cooking loss, and flesh color were determined according to a previously reported method [23]. For the texture profile analysis and shear force measurement, muscle samples were boiled in distilled water for 3 min, dried with absorbent paper, cooled to room temperature, and then subjected to a TA-XT Plus texture analyzer (Stable Micro Systems, Surrey, UK). The hardness, chewiness, cohesiveness, and springiness were recorded via a texture profile analysis. For the shear force, an A/MORS cutter probe (Stable Micro Systems, Surrey, UK) was used with a trigger force of 5 gf and a test speed of 1 mm/s. The second abdominal muscle segment was placed perpendicular to the blade, and the maximum shear force was recorded.

For the cooking loss analysis, approximately 3 g of muscle (denoted as W1) was boiled for 3 min, cooled, dried, and reweighed (W2). The cooking loss was calculated as

Cooking loss (%) = 100 × (W1 − W2)/W1

The flesh color (L*, lightness; a*, redness; b*, yellowness) was measured using an NH310 colorimeter (3nh, Shenzhen, China) after cooking. The muscle samples for the texture profile analysis, shear force, and cooking loss were cooked as described above (boiled for 3 min). The muscle samples for color measurement were also measured after cooking, as the cooked muscle color is the commercially relevant quality attribute. Fresh (raw) muscle samples were used only for the preparation of paraffin sections and H&E staining. All other physicochemical analyses were performed on cooked muscle. This distinction is now clearly indicated for each analysis method. Because all physicochemical measurements were performed on cooked muscle, the results reflect the quality attributes of the cooked product, and extrapolation to raw muscle should be made with caution.

### 2.9. Statistical Analyses

The data are presented in the format of mean ± SD and analyzed using SPSS Statistics software, version 25 (IBM Corp., Armonk, NY, USA). Prior to the ANOVA, the normality of distribution was assessed using the Shapiro–Wilk test, and the homogeneity of variances was verified using Levene’s test. Data that violated the assumptions of normality or homoscedasticity were either log-transformed to meet the parametric assumptions or analyzed using non-parametric alternatives (Kruskal–Wallis test followed by Dunn’s post hoc test). All antioxidant and gene expression data passed the normality and homogeneity tests after log transformation where necessary. Following the one-way ANOVA, Duncan’s multiple range test was used for the post hoc analysis, with statistical significance set at *p* < 0.05. Given the large number of endpoints evaluated, no formal correction for multiple comparisons was applied; this is acknowledged as a limitation, and the results are interpreted accordingly. For data visualization, GraphPad Prism 8.0 (GraphPad Software, Inc. San Diego, CA, USA) was employed.

## 3. Results

### 3.1. The H&E Staining of Prawn Intestine

**Figure 1:**
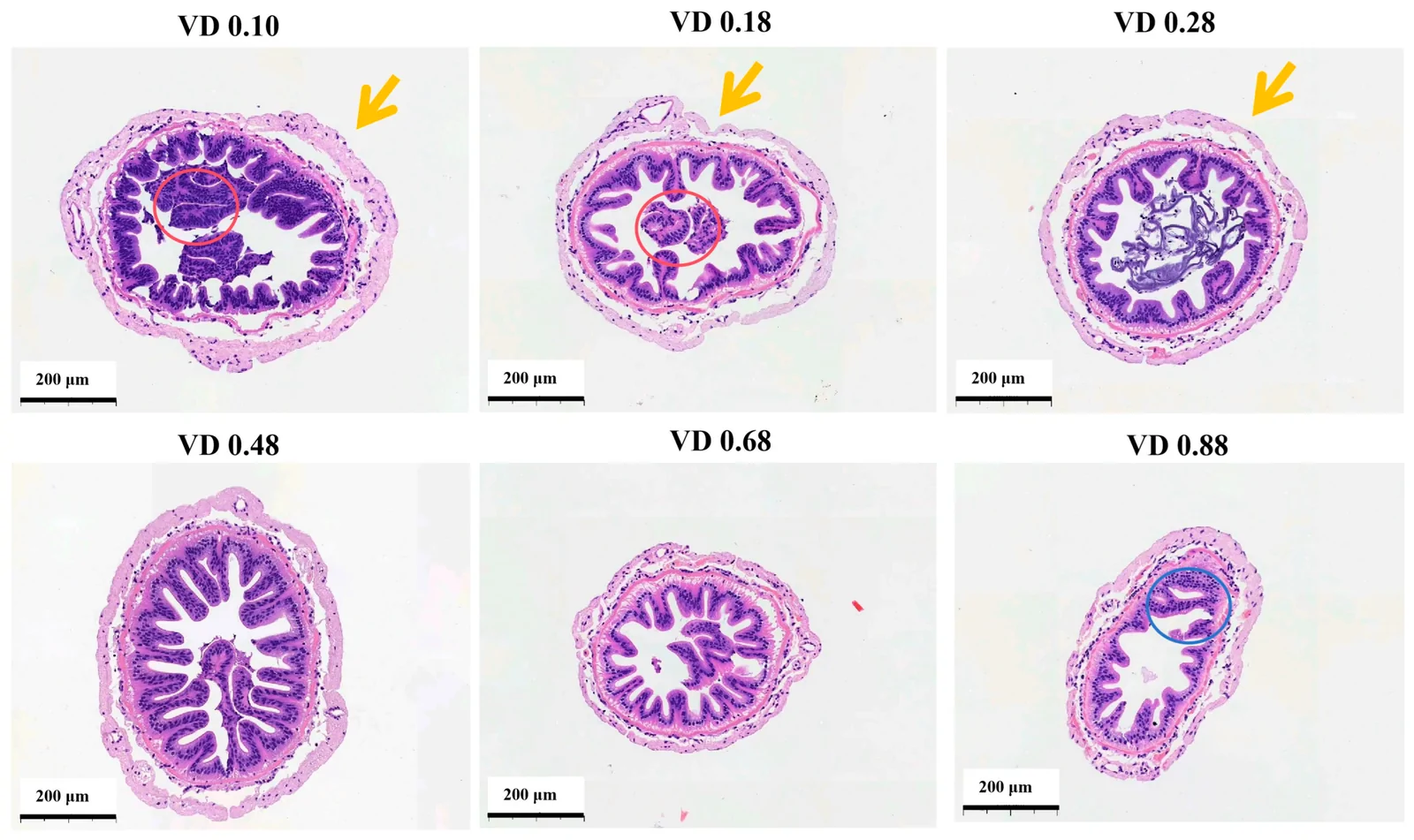
Effects of VD_3_ on intestinal histology of *M. rosenbergii* after non-O1 *Vibrio cholerae* challenge (400×; *n* = 3). Yellow arrows indicate massive inflammatory infiltration; red circles indicate disintegrated mucosal villi; blue circle indicates disarranged epithelial cells.

H&E staining was performed to evaluate the histopathological changes in the intestine of *M. rosenbergii* among groups with different VD_3_ levels (Figure 1). The VD 0.10 group displayed histopathological damage, where the entire intestinal structure was destroyed and normal mucosal villi had disintegrated, accompanied by edema and pronounced inflammatory infiltration in the thickness of the intestinal wall. The VD 0.18 group showed intestinal injury, with collapsed mucosal villus structure, necrosis and exfoliation of epithelial cells. The VD 0.28-VD 0.68 groups included retained an overall intact intestinal architecture, with only a slight reduction in villus height, and rare apical epithelial exfoliation. The VD 0.88 group exhibited intestinal injury, characterized by villus atrophy and shortening, disarrangement of intestinal epithelial cells, and a small amount of inflammatory cell infiltration in the submucosa and muscularis.

### 3.2. The H&E Staining of Prawn Hepatopancreas

**Figure 2:**
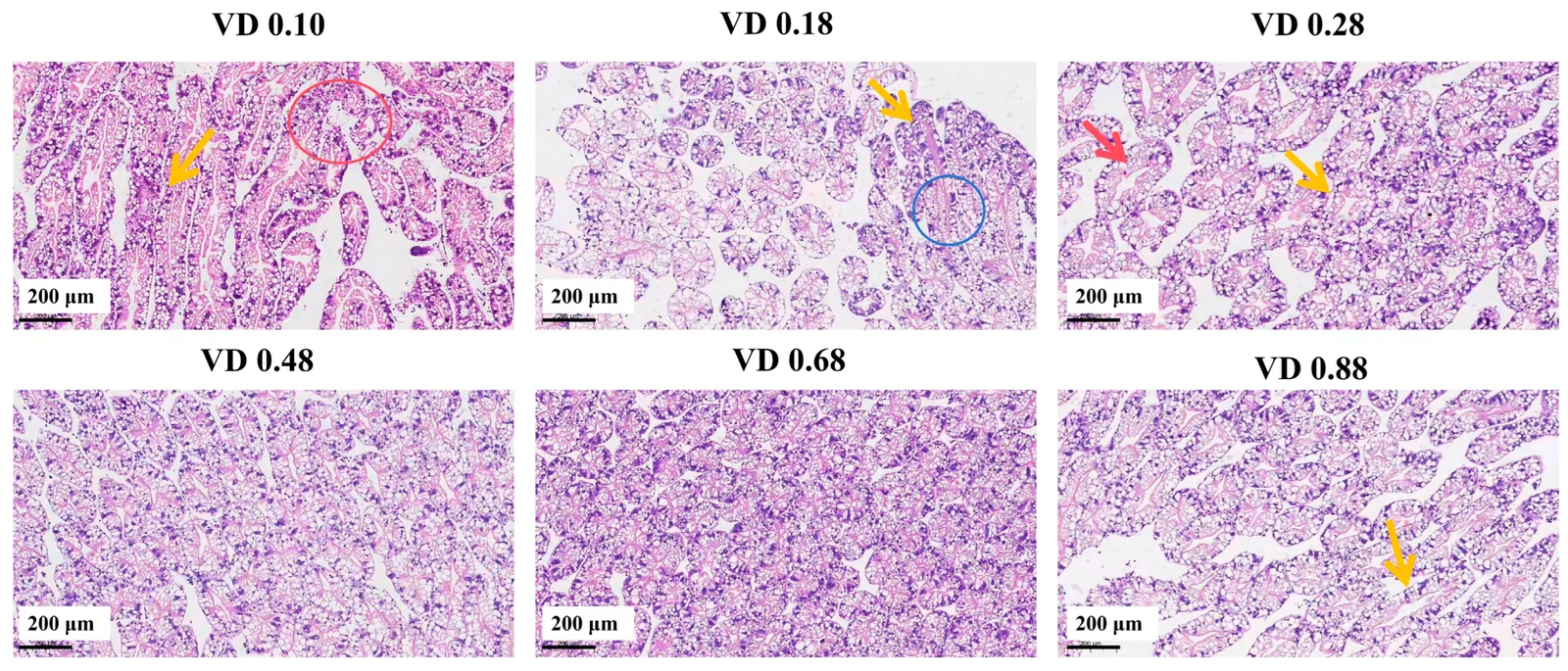
Effects of VD_3_ on hepatopancreas histology of *M. rosenbergii* after non-O1 *Vibrio cholerae* challenge (200×; *n* = 3). Yellow arrows indicate elongated and distorted tubules; red circle indicates extensive vacuolar degeneration; blue circle indicates exfoliation and structural loss; red arrows indicate dilated and deformed tubules.

As shown in Figure 2, histopathological observation of *M. rosenbergii* hepatopancreas revealed graded structural alterations among the groups following dietary VD_3_ supplementation. The tubules of VD 0.10 group were elongated, distorted, and fragmented, accompanied by massive epithelial necrosis and extensive vacuolar degeneration. The VD 0.18 group exhibited disorganized tubules, widespread epithelial vacuolization and exfoliation, and partial loss of normal tubular structure. The VD 0.28 and VD 0.88 groups displayed comparable moderate pathological alterations: their tubules were loosely organized with obvious dilation and deformation, the intertubular spaces were expanded, and the epithelial cells presented with marked vacuolar degeneration. The VD 0.48 and 0.68 group exhibited an intact hepatopancreatic architecture, characterized by densely packed, regularly arranged tubules with round or oval cross-sections, and preserved epithelial cells.

### 3.3. The Enzymes of the Intestine and Hepatopancreas Tissues

**Figure 3:**
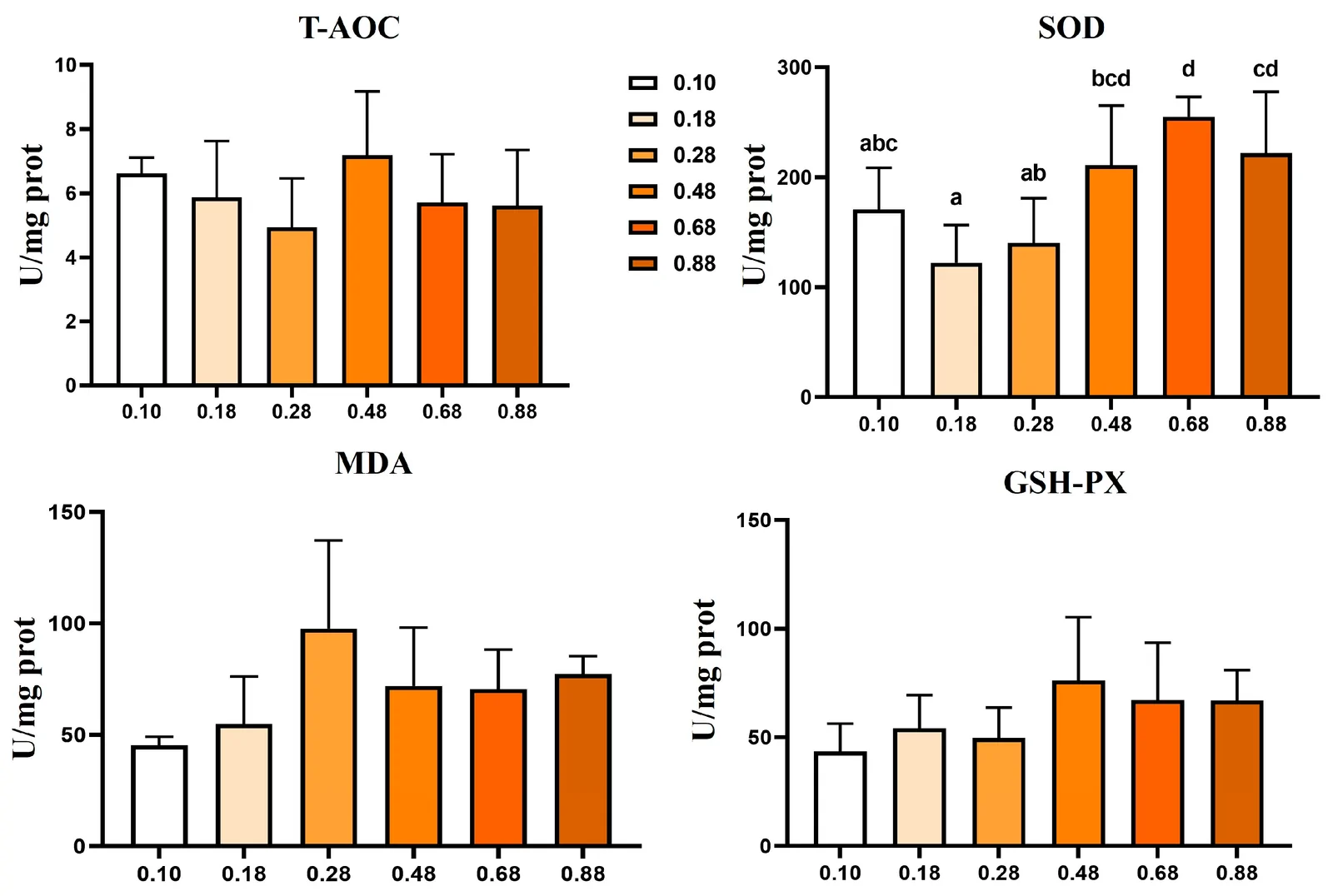
Effects of VD_3_ on MDA content and enzyme activities of antioxidant parameters in prawn intestine after non-O1 *Vibrio cholerae* challenge (*n* = 3). Values are shown as means ± SD. Results with different superscript letters are significantly different (*p* < 0.05). MDA, malondialdehyde; T-AOC, total antioxidant capacity; SOD, superoxide dismutase; GSH-PX, glutathione peroxidase.

**Figure 4:**
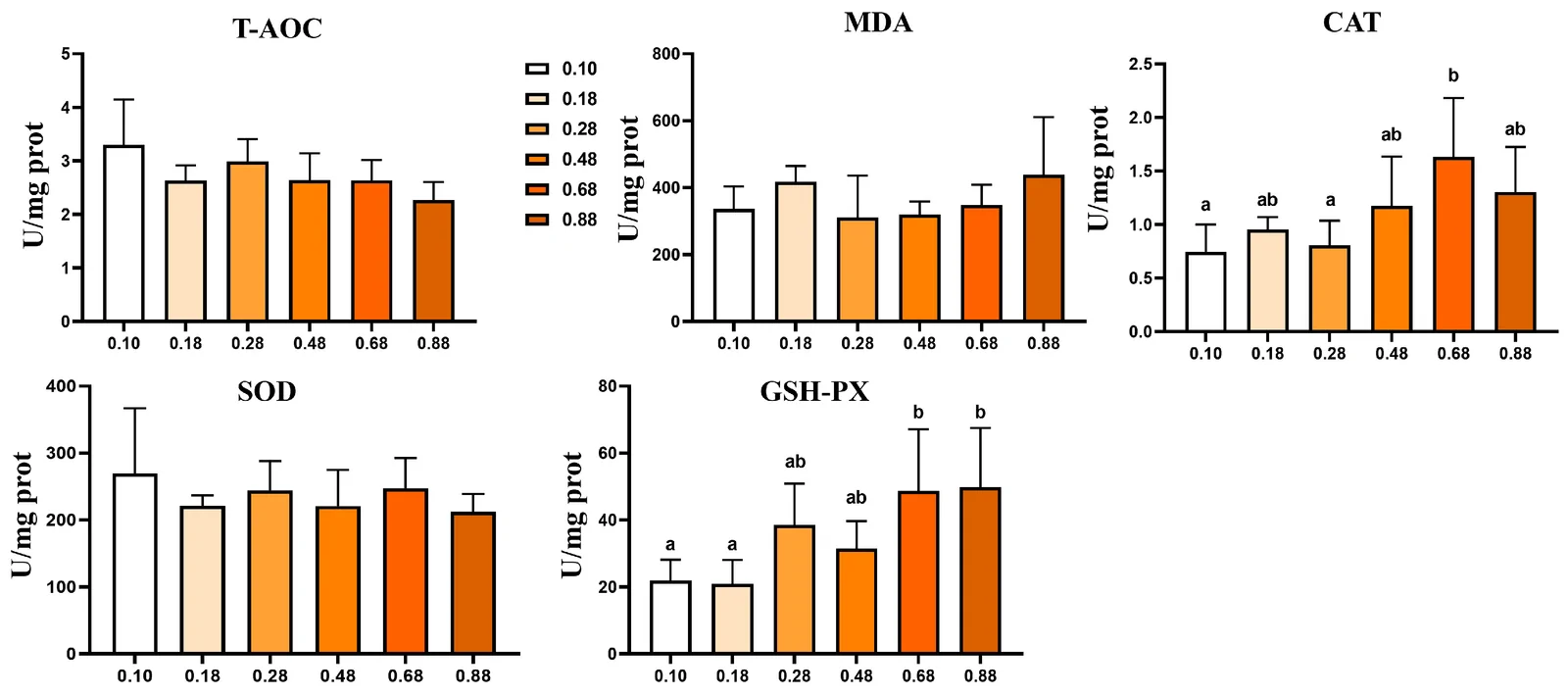
Effects of VD_3_ on MDA content and enzyme activities of antioxidant parameters in prawn hepatopancreas after non-O1 *Vibrio cholerae* challenge (*n* = 3). Values are shown as means ± SD. Results with different superscript letters are significantly different (*p* < 0.05). MDA, malondialdehyde; SOD, superoxide dismutase; CAT, catalase; GSH-PX, glutathione peroxidase; T-AOC, total antioxidant capacity.

The antioxidant responses to the dietary VD_3_ were tissue-specific and parameter-dependent. In the intestine, only the SOD activity showed a significant response to VD_3_ supplementation, while the T-AOC, MDA, and GSH-PX remained unchanged (*p* > 0.05); in the hepatopancreas, the CAT and GSH-PX activities increased significantly, whereas the SOD, MDA, and T-AOC showed no significant differences (*p* > 0.05). The SOD activity showed an increasing trend with dietary VD_3_ supplementation and was significantly higher in the VD 0.68 group compared with the control. There were no significant differences in the indices of T-AOC, MDA, and GSH-PX among all the groups (Figure 3; *p* > 0.05). The CAT activity in the hepatopancreas increased with the VD_3_ level. Compared with the other treatments, the highest CAT activity was recorded in the VD 0.68 group. The activity of GSH-PX was also significantly affected by VD_3_ supplementation **(**Figure 4; *p* < 0.05). The GSH-PX activity was enhanced with an increasing VD_3_ addition, and the VD 0.68 and VD 0.88 groups showed the highest levels compared with the other groups. These were no significant differences in the MDA content or in the activities of SOD and T-AOC in the hepatopancreas (*p* > 0.05).

### 3.4. The Gene Expression of the Intestine and Hepatopancreas Tissues

**Figure 5:**
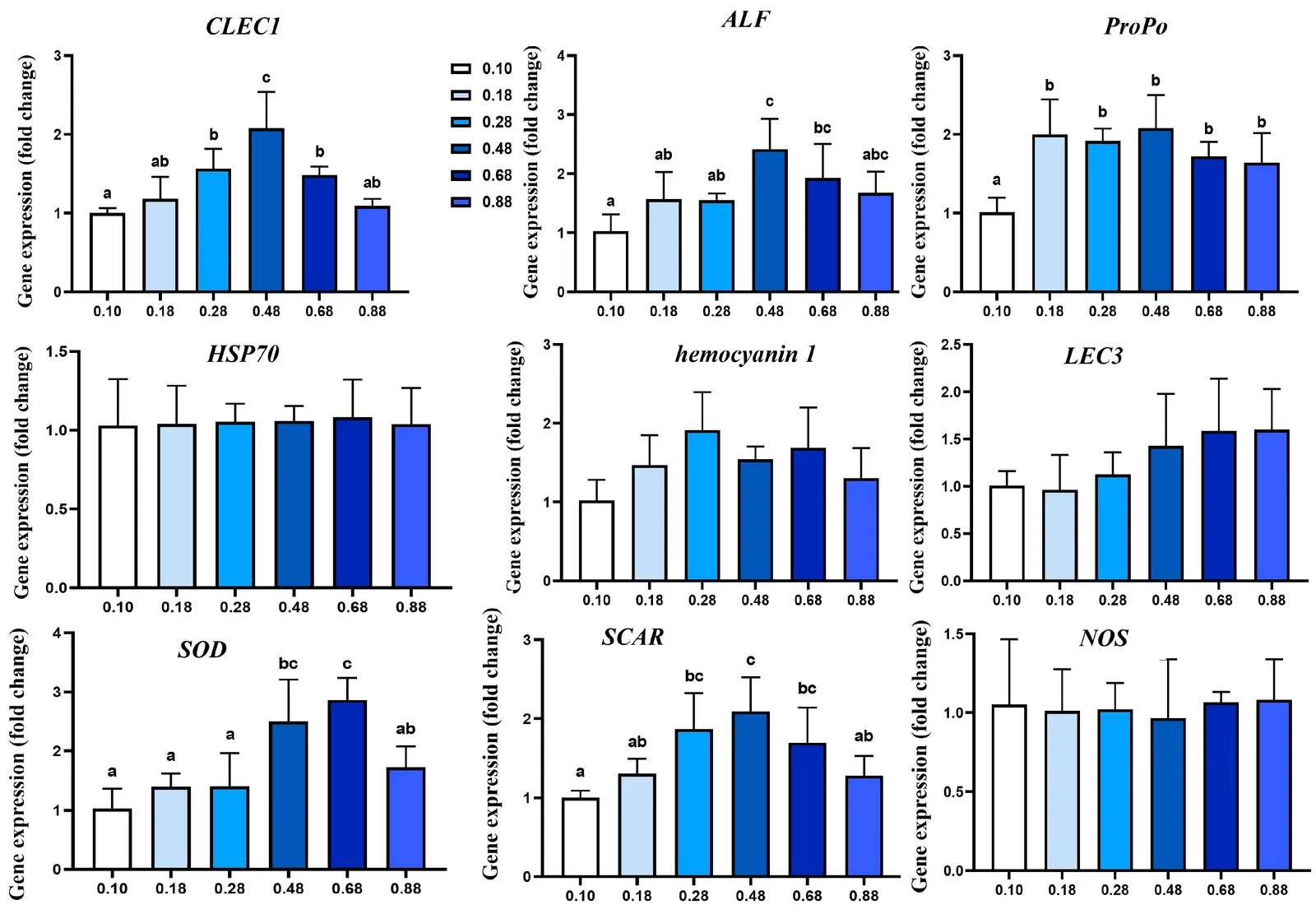
Effects of VD_3_ on gene levels of immune function in prawn intestine after non-O1 *Vibrio cholerae* challenge (*n* = 3). Values are shown as means ± SD. Results with different superscript letters are significantly different (*p* < 0.05). CLECT, c-type lectin; HSP70, heat shock protein 70; *ALF*, anti-lipopolysaccharide factor; SOD, superoxide dismutase; ProPO, prophenoloxidase; SCAR, scavenger receptor class; NOS, nitric oxide synthase; LEC3, lectin 3.

In the intestine, the CLECT gene expression increased with increasing dietary VD_3_ levels, reaching the highest value in the VD 0.48 group, which was 2.08-fold that of the control group (VD 0.10) (Figure 5; *p* = 0.002); its expression in the VD 0.28 (1.56-fold) and VD 0.68 (1.49-fold) groups was also significantly higher than in the control (*p* < 0.05). The ALF mRNA level peaked in the VD 0.48 group, at 2.42-fold that of the control (*p* = 0.029). The SOD gene expression increased with enhanced VD_3_ supplementation, and the highest level was observed in the VD 0.68 group (2.86-fold that of the control; *p* = 0.002), followed by the VD 0.48 group (2.50-fold). The ProPO expression in all VD_3_-supplemented groups was significantly increased by 1.64–2.08-fold compared with the control group (*p* = 0.015). The SCAR mRNA level gradually increased with VD_3_ supplementation, peaking in the VD 0.48 group at 2.09-fold that of the control (*p* = 0.017). There were no significant differences in the expression of hemocyanin 1, HSP70, NOS, or LEC3 among the groups (*p* > 0.05).

**Figure 6:**
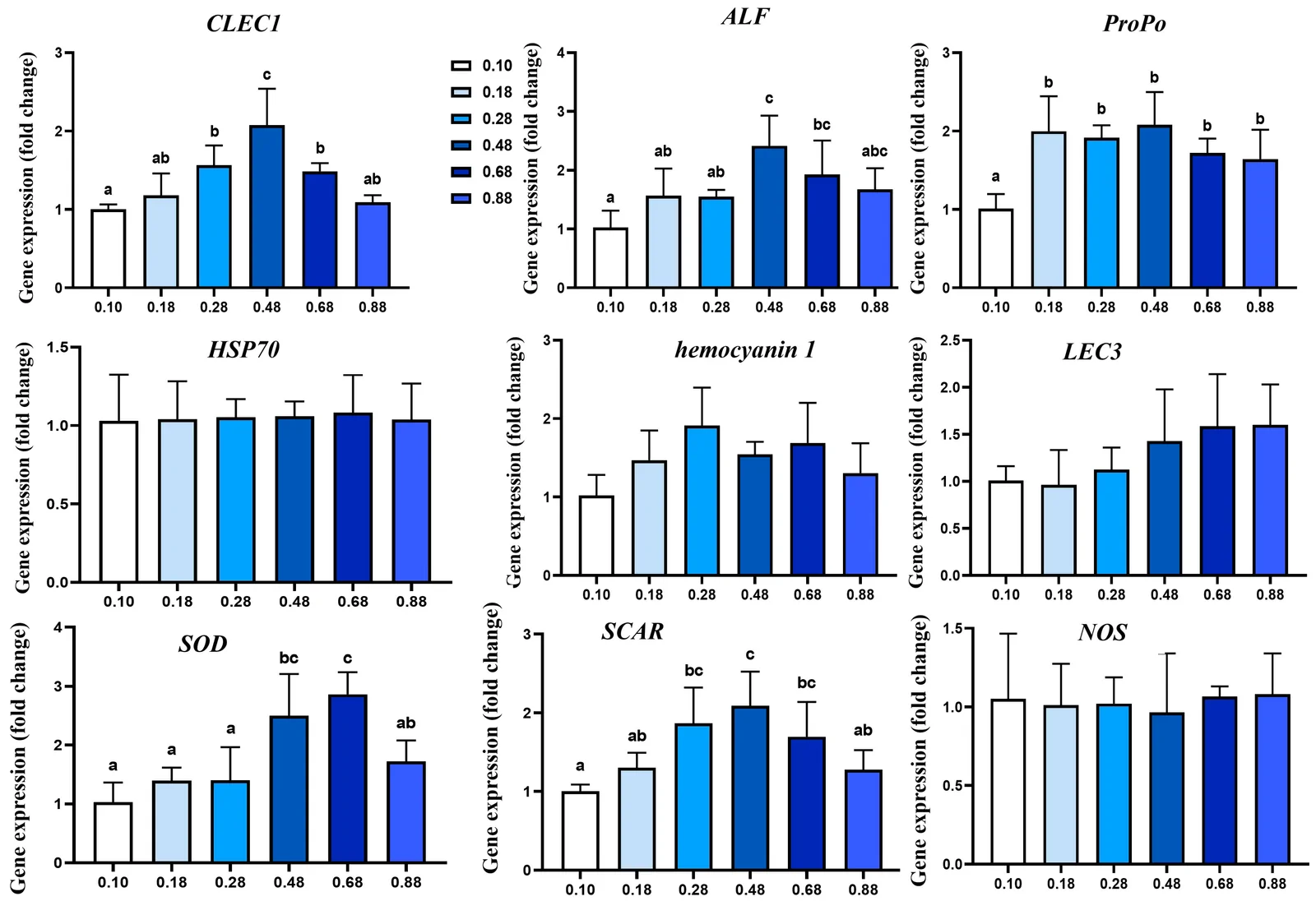
Effects of VD_3_ on gene levels of immune function in the prawn hepatopancreas after non-O1 *Vibrio cholerae* challenge (*n* = 3). Values are shown as means ± SD. Results with different superscript letters are significantly different (*p* < 0.05). CLECT, c-type lectin; LEC3, lectin 3; *ALF*, anti-lipopolysaccharide factor; SCAR, scavenger receptor class; SOD, superoxide dismutase; NOS, nitric oxide synthase; SERPIN 1, serine protease inhibitor 1.

In the hepatopancreas, the CLECT mRNA level increased with VD_3_ supplementation and reached the highest level in the VD 0.48 group, at 2.29-fold that of the control group (*p* = 0.026); the VD 0.28 (2.09-fold) and VD 0.68 (2.18-fold) groups were also significantly higher than the control. The mRNA level of SOD gradually increased with dietary VD_3_ addition, and the highest expression was recorded in the VD 0.48 group (2.90-fold that of the control; *p* = 0.003). The VD 0.48 group achieved the highest level of SCAR gene expression compared with the other treatments, at 2.56-fold that of the control (Figure 6; *p* = 0.001). There were no significant differences in the expression of HSP70, hemocyanin 1, LEC3, or NOS among the groups (*p* > 0.05).

### 3.5. The Histology and Physicochemical Properties of the Prawn Muscle

**Figure 7:**
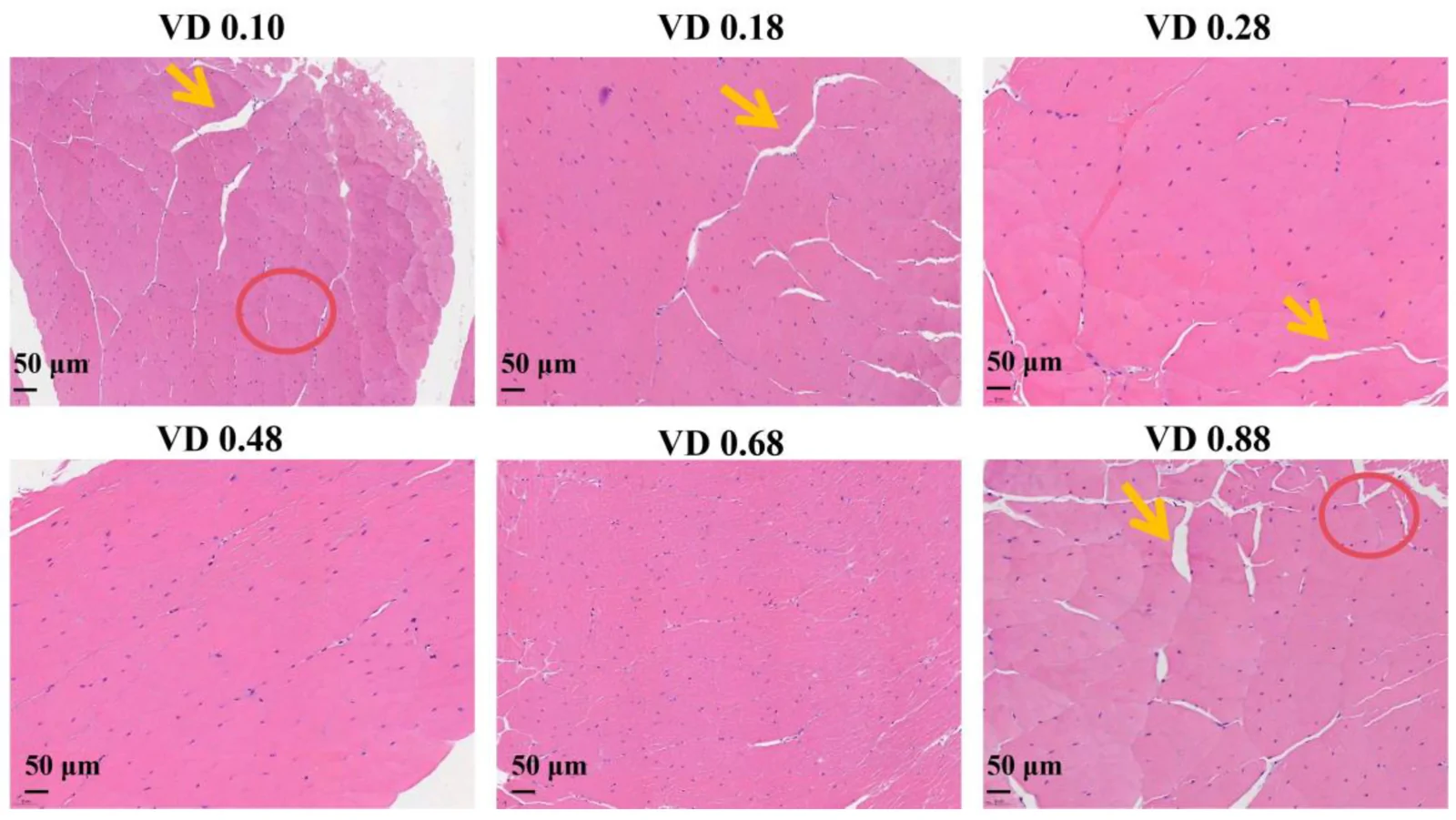
Effects of VD_3_ on muscle histology of *M. rosenbergii* after non-O1 *Vibrio cholerae* challenge (*n* = 3; H&E staining, 200×; raw muscle samples). Yellow arrows indicate widened intermuscular space and red circles indicate disordered myofiber arrangement.

No severe histopathological lesions were observed in the muscles of prawns across all treatment groups after the bacterial challenge (Figure 7). The prawns in the control group (VD 0.10) showed slight structural alterations, including disordered myofiber arrangement and widened intermuscular space caused by edema. Muscle sections from the VD 0.48 and VD 0.68 groups showed more regular myofiber arrangement compared with the control group. However, excessive VD_3_ supplementation (0.88 mg/kg) diminished the above beneficial effects, with reappearance of widened intermuscular space and disordered myofiber arrangement.

**Figure 8:**
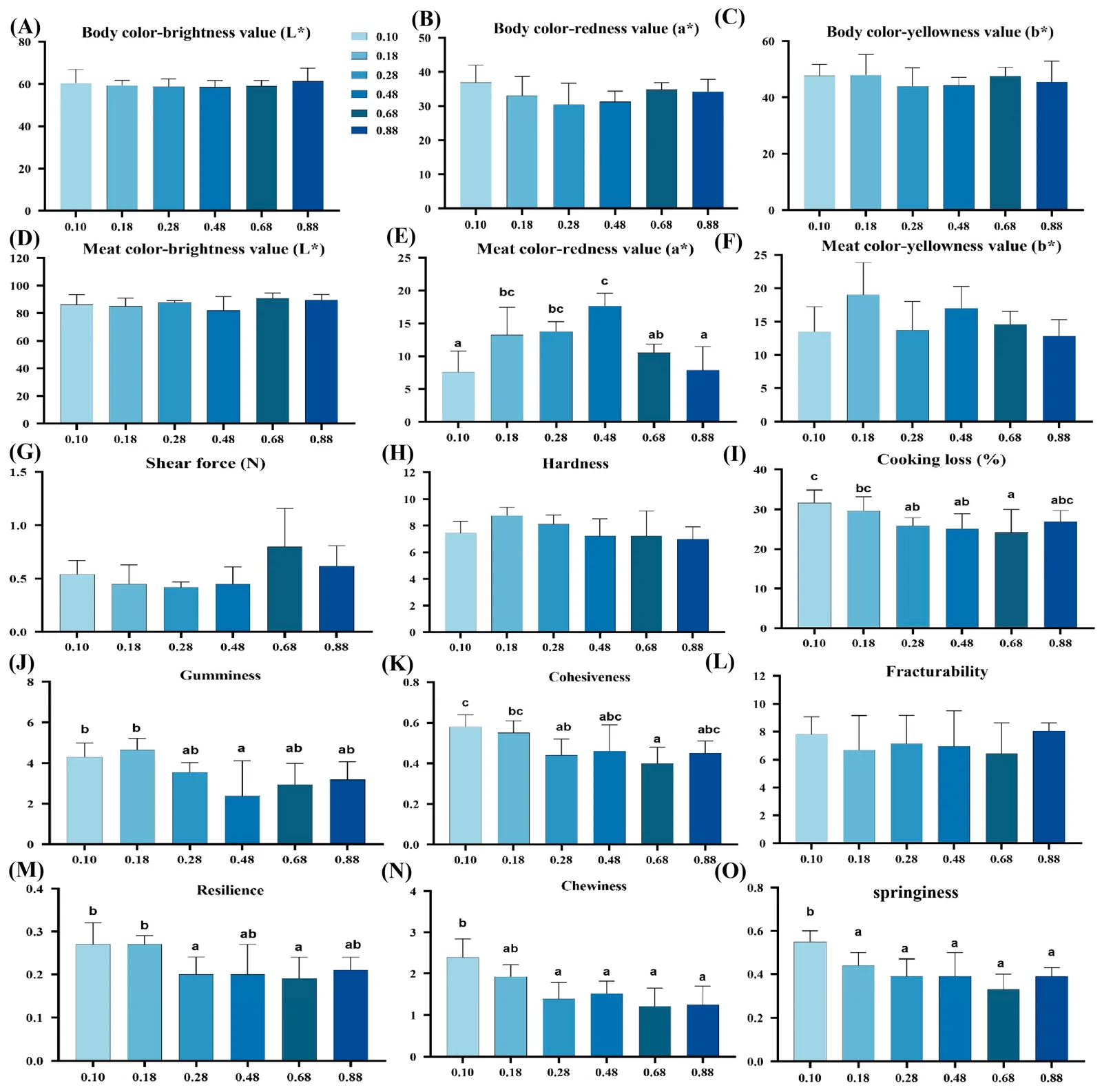
Effects of VD_3_ on the physicochemical properties of prawn muscle after non-O1 *Vibrio cholerae* challenge. (**A**) L*, value of body color; (**B**) a*, value of body color; (**C**) b*, value of body color; (**D**) L*, value of meat color; (**E**) a*, value of meat color; (**F**) b*, value of meat color; (**G**) shear force; (**H**) hardness; (**I**) cooking loss; (**J**) gumminess; (**K**) cohesiveness; (**L**) fracturability; (**M**) resilience; (**N**) chewiness; (**O**) springiness. (**A**–**C**) Body color of cooked prawns; (**D**–**F**) meat color of cooked muscle; (**G**–**O**) textural properties of cooked muscle. Results are shown as means ± SD (*n* = 3). Different letters above bars indicate significant differences (*p* < 0.05).

Figure 8 depicts the impacts of graded dietary VD_3_ concentrations on body color, meat color, and textural properties of the prawn muscle. With increasing VD_3_ levels, the brightness value, redness value and yellowness value in body color exhibited gradual changes, but no significant differences were observed across the different groups (*p* > 0.05). The meat brightness and yellowness had no significant differences among the different VD_3_ levels (*p* > 0.05). The meat redness differed significantly among groups; the 0.48 group had the highest level among all treatments (*p* < 0.05). For the textural indices, the VD 0.10 group had the highest cooking loss, and the lowest value was in the VD 0.68 group. The gumminess of VD 0.48 group was significantly decreased compared with the control group (*p* < 0.05). Compared with the VD_3_ supplement group, VD 0.68 achieved the lowest value for cohesiveness. The resilience was higher in the 0.10–0.18 groups and then decreased in the 0.28–0.88 groups. The VD_3_ supplement levels (0.28–0.88) were significantly decreased compared with the control group (*p* < 0.05). The value for springiness was higher in the 0.10 group and then significantly reduced in the 0.18–0.88 groups (*p* < 0.05). No significant differences were detected across all VD_3_ supplementation groups in the shear force, hardness or fracturability, although the springiness, gumminess, cohesiveness, and resilience were significantly lower in the VD_3_-supplemented groups than in the control (*p* < 0.05).

## 4. Discussion

VD_3_ is an essential fat-soluble micronutrient that regulates multiple physiological processes in animals, including calcium metabolism, antioxidant defense, and immune modulation [11]. The dietary supplementation of functional nutrients to improve health status and product quality has become a research focus in aquaculture nutrition. The present study systematically investigated the effects of dietary VD_3_ on antioxidant capacity, immune-related gene expression, and muscle physicochemical properties in prawns, with a focus on tissue-specific responses. The findings provide additional evidence that dietary VD_3_ may influence physiological responses in *M. rosenbergii* under bacterial challenge.

### 4.1. Effects of Dietary VD_3_ on Intestinal and Hepatopancreatic Histopathology in M. rosenbergii Post Non-O1 Vibrio cholerae Challenge

The intestine and hepatopancreas are the primary target organs of Vibrio infection in crustaceans, and their structural integrity is the prerequisite for maintaining normal functions and disease resistance [26,27]. In the present study, dietary VD_3_ supplementation was associated with reduced histological alterations. The prawns fed the diet with 0.68 mg/kg VD_3_ maintained nearly normal intestinal and hepatopancreatic histological structures, with intact mucosal villi, tightly arranged epithelial cells, and regularly shaped hepatopancreatic tubules. Consistent with our histopathological observations, previous evidence has also revealed that prawns fed a basal diet with moderate dietary VD_3_ had markedly elevated intestinal villus height and muscular layer thickness, and maintained intact and well-organized intestinal mucosal architecture [12,28]. By contrast, excessive VD_3_ addition diminished the above beneficial morphological improvements under high salinity stress [17]. Dietary VD_3_ supplementation effectively ameliorated histopathological damage induced by *Nocardia seriolae* infection, reduced pathogen loads, and mitigated cellular necrosis in liver of largemouth bass (*Micropterus salmoides*). The protective effects of VD_3_ on tissue structure may be attributed to an enhanced antioxidant capacity and innate immunity under pathogenic challenge. The tissue-protective association of VD_3_ may be partly explained by an enhanced antioxidant capacity and innate immune gene expression under pathogenic challenge, although contributions from improved nutritional status, altered feed intake, and growth- or metabolism-related effects cannot be excluded. Therefore, we further investigated the effects of VD_3_ on antioxidant capacity and innate immunity.

### 4.2. Effects of Dietary VD_3_ on Antioxidant Capacity in M. rosenbergii Post Bacterial Challenge

Bacterial infection typically induces a strong oxidative stress response in crustaceans, leading to the excessive production of ROS, which can cause oxidative damage to lipids, proteins, and nucleic acids, and ultimately result in tissue injury [29]. To counteract oxidative stress, crustaceans have evolved an enzymatic antioxidant defense system, mainly consisting of SOD, CAT, and GSH-PX, which work synergistically to maintain redox homeostasis [30]. In the present study, dietary VD_3_ supplementation was associated with altered antioxidant responses in both the hepatopancreas and intestine of *M. rosenbergii* after a non-O1 *Vibrio cholerae* challenge. In the hepatopancreas, the activities of CAT and GSH-PX increased with rising dietary VD_3_ levels, with the highest values observed in the 0.68–0.88 mg/kg VD_3_ groups. In the intestine, SOD activity showed an increasing trend with dietary VD_3_ supplementation and was significantly higher in the VD 0.68 group compared with the control. Previous investigations on *Litopenaeus vannamei* further verified the conserved antioxidant regulatory effects of VD family metabolites in crustaceans. Dietary supplementation with 25-OH-D_3_ significantly elevated the activities of SOD, CAT, and T-AOC in both the hemolymph and hepatopancreas [18]. Similarly, studies in grass carp (*Ctenopharyngodon idella*) demonstrated that VD_3_ supplementation enhanced intestinal antioxidant enzyme activities and alleviated lipid peroxidation induced by oxidative stress [12]. VD_3_ in the diet increased the activities of SOD, CAT, and GPx, and decreased the MDA content in *P. vannamei* [31]. In different salinity conditions, VD_3_ enhanced the gene expression of SOD and CAT, and decreased the MDA expression level in the hepatopancreas of *L. vannamei*. These results strongly support our present data that appropriate dietary VD_3_ supplementation enhanced selected components of the antioxidant defense system, although this did not result in measurable changes in lipid peroxidation. Notably, we observed tissue-specific differences in the antioxidant response to VD_3_: the intestine showed a more pronounced response to SOD activity. This tissue-specific difference may be explained by the fact that the intestine, as the frontline organ directly exposed to dietary components and invading pathogens, is more susceptible to oxidative stress during bacterial infection [32]. The stronger SOD response observed in the intestine may reflect tissue-specific regulation. VD_3_ modulates distinct antioxidant pathways in different organs rather than uniformly enhancing the global antioxidant capacity. The lack of significant changes in the MDA content across all groups suggests that lipid peroxidation levels were not substantially altered by VD_3_ supplementation under the experimental conditions, possibly because the 72 h post-challenge time point was too early to detect pronounced lipid peroxidation differences, or because other compensatory mechanisms maintained redox homeostasis.

### 4.3. Regulatory Effects of Dietary VD_3_ on Immune-Related Gene Expression in M. rosenbergii

Crustaceans lack an adaptive immune system and rely entirely on innate immunity to defend against pathogenic infection [8]. The innate immune system of crustaceans mainly consists of pattern recognition receptors, antimicrobial peptides, the prophenoloxidase activating system, and cellular immune responses such as phagocytosis [7]. In the present study, we found that dietary VD_3_ supplementation significantly modulated the mRNA expression levels of multiple immune-related genes in the intestine and hepatopancreas of *M. rosenbergii* after a non-O1 *Vibrio cholerae* challenge. In the intestine, VD_3_ supplementation similarly upregulated the expression of pattern recognition receptor genes (*CLECT* and *SCAR*), antimicrobial peptides (*ALF*), immune regulatory genes, and *ProPo*. In the hepatopancreas, VD_3_ supplementation significantly upregulated the expression of *CLECT*, *SCAR*, *SOD*, and *NOS*. Moderate VD_3_ supplementation significantly elevated the mRNA abundance of key immune effector genes in the hepatopancreas of *L. vannamei*, including *ProPo*, lysozyme, alkaline phosphatase and acid phosphatase [18]. VD_3_ increased the non-specific immunity in the hemolymph of Pacific white shrimp (*P. vannamei*) [33]. Consistent with our present results, dietary VD_3_ supplementation markedly elevated the mRNA level of ALF in crustaceans across different salinity environments [17]. The upregulated immune-related gene expression suggests a potential immunomodulatory effect of VD_3_ at the transcriptional level in freshwater prawns under a bacterial challenge; whether these transcriptional changes translate into enhanced immune function requires further study. Tissue-specific response patterns of immune-related genes to dietary VD_3_ were observed in this study. For the antimicrobial peptide *ALF*, significant inductive expression was detected in the intestine, while no significant change was found in the hepatopancreas. This discrepancy can be attributed to the functional division of the two organs: the intestine, as the first mucosal barrier against pathogens, is the primary synthesis site of mucosal antimicrobial peptides [34]. In contrast, NOS, which mediates systemic immune regulation and oxidative stress homeostasis [30], showed a significant dose-dependent response in the hepatopancreas but not in the intestine. This is consistent with the fact that the hepatopancreas is the core detoxification organ of crustaceans, where inducible NOS is mainly expressed in response to pathogen invasion and oxidative stress [35]. The reduced responses observed at the highest VD_3_ level suggest a possible optimal supplementation range; however, the mechanisms underlying this response require further investigation.

### 4.4. Effects of Dietary VD_3_ on Muscle Physicochemical Properties of M. rosenbergii

Flesh quality is a key economic trait of farmed prawns, which directly affects consumer acceptability and market value [22]. In the present study, we found that dietary VD_3_ supplementation modulated certain muscle physicochemical parameters of *M. rosenbergii*. In terms of flesh color, the prawns fed the diet with 0.48 mg/kg VD_3_ had a significantly higher redness value (a*) than the other groups, while no significant differences were observed in brightness (L*) or yellowness (b*) values across all groups. Flesh redness is a critical quality attribute that is positively correlated with consumer preference. In crustaceans, muscle redness is primarily determined by carotenoid pigments, especially astaxanthin and carotenoproteins (crustacyanin), rather than myoglobin [36,37]. For cooking loss, the 0.68 mg/kg VD_3_ group showed the lowest cooking loss among all groups. The reduction in cooking loss with VD_3_ supplementation may be due to its antioxidant effects: the reduced cooking loss may indicate improved water retention properties, although the underlying mechanism remains unclear. The significantly reduced cooking loss is another key driver of the modulated textural properties. Reduced cooking loss increases the bound water content in muscle fibers, reduces the friction between fiber bundles, and thus decreases the chewiness, springiness and resilience of the muscle [38]. The effects of VD_3_ on flesh quality involve both constitutive nutritional enhancement and challenge-responsive modulation [23]. VD_3_ supplementation altered several texture parameters, although the relationship with consumer-perceived quality requires further evaluation.

Several limitations of this study should be acknowledged. First, VD metabolism and VDR-mediated signaling were not directly measured, so the mechanisms underlying the observed responses remain speculative. Second, immune responses were assessed only at the transcriptional level, without functional immune assays (e.g., phenoloxidase activity, phagocytosis, or antibacterial activity). Third, only one pathogen was used in the challenge experiment, and sampling was conducted at a single 72 h time point. Finally, all flesh quality measurements were performed on cooked muscle, which limits the extrapolation to raw muscle quality.

## 5. Conclusions

Taken together, the results of the present study demonstrate that dietary VD_3_ supplementation modulated several physiological responses of *M. rosenbergii* challenged with non-O1 *Vibrio cholerae*, including alleviating histopathological damage in the intestine and hepatopancreas, modulating antioxidant enzyme activities in a tissue-specific manner, upregulating the mRNA expression of certain immune-related genes, and altering selected muscle physicochemical properties. Based on the comprehensive evaluation of all tested indicators, the dietary VD_3_ levels of 0.48–0.68 mg/kg showed the most favorable overall responses among the tested treatments (Supplementary Figure S1). This range is based on both a descriptive evaluation and formal dose–response modeling, while acknowledging that some parameters peaked at different doses within this range. These findings highlight the potential of VD_3_ as a functional feed additive that may enhance the physiological responses associated with host defense and product quality in *M. rosenbergii* farming, and provide preliminary evidence supporting the further evaluation of VD_3_ as a functional feed additive. Further studies are required to determine the long-term effects and validate the optimal supplementation level under commercial farming conditions.

## Data Availability

The datasets are included in this article and available from the corresponding author on reasonable request.

## Supplementary Materials

The following supporting information can be downloaded at: https://www.mdpi.com/article/10.3390/ani16162463/s1. Table S1: Ingredient compositions of experimental diets. Table S2: Method for analysis of biomarker and enzymes activity-related parameters. Table S3: Real-time PCR primer sequences. Figure S1: One-way ANOVA and polynomial (quadratic) regression analysis of different muscle parameters.

## Abbreviations

ALF	anti-lipopolysaccharide factor
CAT	catalase
cDNA	complementary deoxyribonucleic acid
CLECT	c-type lectin
cfu/mL	colony-forming units per milliliter
EF-1α	elongation factor 1α
GSH-PX	glutathione peroxidase
H&E	hematoxylin & eosin
HSP70	heat shock protein 70
LEC3	lectin 3
MDA	malondialdehyde
NOS	nitric oxide synthase
ProPO	prophenoloxidase
qPCR	real-time quantitative polymerase chain reaction
ROS	reactive oxygen species
SCAR	scavenger receptor SD, standard deviation
SERPIN 1	serine protease inhibitor 1
SOD	superoxide dismutase
T-AOC	total antioxidant capacity
VD_3_	vitamin D_3_
