# Supplementary material for: Dietary Vitamin D3 Modulates Antioxidant Responses, Immune-Related Gene Expression, and Flesh Quality in Giant Freshwater Prawn (Macrobrachium rosenbergii) Under Pathogenic Vibrio Challenge
Source: Animals (Basel). 2026 Aug 7;16(16):2463. doi: 10.3390/ani16162463 (PMC13508868; doi:10.3390/ani16162463)
Supplement: Supplementary file 1 [file animals-16-02463-s001.zip › animals-4392244-supplementary.pdf]

Supplementary Table S1. Ingredient compositions of experimental diets.

Ingredients (g/kg)		Nutrients content (%)	
Fish meal	280.00	Moisture	9.17
Soybean meal	200.00	Crude protein	40.05
Wheat	220.00	Crude fat	9.26
Soybean protein concentrate	115.00	Ash	10.30
Shrimp powder	50.00	Gross energy (KJ/g)	18.08
Linseed oil	25.00		
Soy oil	36.00		
Cholesterol	4.00		
Choline chloride (50%)	10.00		
Vitamin premix [1]	1.00		
Mineral premix [1]	2.50		
Corn starch	16.50		
Vitamin D_3 (VD_3) premix [2]	10.00		
Zeolite powder	10.00		
$Ca(H_2PO_4)_2$	20.00		
Total	1000.00		

[1] Vitamin (IU or mg/kg) and mineral mix (g kg-1) were as follows: $CuSO_4 \cdot 5H2O$, 2.0 g; $FeSO_4 \cdot 7H2O$, 25 g; $ZnSO_4 \cdot 7H2O$, 22 g; $MnSO_4 \cdot 4H2O$, 7 g; Na_2SeO_3, 0.04 g; KI, 0.026 g; $CoCl_2 \cdot 6H2O$, 0.1 g; vitamin A, 900,000 IU; vitamin E, 4500 mg; vitamin K_3, 220 mg; vitamin B_1, 320 mg; vitamin B_2, 1090 mg; vitamin B_5, 2000 mg; vitamin B_6, 500 mg; vitamin B_{12}, 1.6 mg; vitamin C, 5000 mg; pantothenate, 1000 mg; folic acid, 165 mg.

[2] VD_3 premix: Premix was added to obtain graded levels of VD_3 and the amount of maize starch was reduced to compensate.

[3] Shrimp powder: Produced from *Euphausia superba* and purchased from Jiangsu Fu Yuda Grain Products Co., Ltd. It is manufactured through cooking and high-temperature drying, which inactivates pathogens.

Supplementary Table S2. Method of analysis for biomarker and enzymes activity-related parameters.

Indices	Analysis method	Catalog number	Detection range
SOD	Water-soluble tetrazolium salt-1 (WST-1 method) assay kit (Nanjing Jiancheng Institute, China)	A001-3-2	0.5-122.1 U/ml
GSH-Px	Colorimetric method assay kit (Nanjing Jiancheng Institute, China)	A005-1-2	20-330U
MDA	Thiobarbituric acid (TBA) assay kit (Nanjing Jiancheng Institute, China)	A003–1	0-113.0 nmol/mL
CAT	Visible light, commercial kit (Nanjing Jiancheng Institute, China)	A007-1-1	0.2-24.8 U/mL
T-AOC	Microplate method (Nanjing Jiancheng Institute, China)	A015-2-1	0.2-55.2 U/ml

[1] CAT, catalase; GSH-PX, glutathione peroxidase; MDA, malondialdehyde; SOD, superoxide dismutase; T-AOC, total antioxidant capacity.

Supplementary Table S3. Real-time PCR primer sequences.

Target Gene[1]	Primer Sequence Forward (5¢→ 3¢)	Primer Sequence Reverse (5¢ → 3¢)	Accession Number	Amplicon (bp)
sod	CTGGGCGTTCGTGGACAT	GGAAAGACGGAAAGGGTC	XM_067086834	102
clec1	GCGTCGTTGAAATGCGATGT	TGACCTCTTAGCGTCTCG	XM_067118292.1	85
hsp70	GCGGAACAGGTCAGAGCA	GGACATCAGCGGAAACAA	XM_067088554.1	181
alf2	GTAGTATTAGATGGGATTTACC	AACAAGGCAGTCCAAGAT	XM_067134113.1	177
propo	GCAACATTGGCGAACTGA	GGGAAGGTCTCGACGACT	XM_067101054	146
scar	GTCAGGGAGGTGGAGGTGA	CTGGGCGTGGACTGGTTT	XM_067082392.1	137
serpin1	CCAGGACCTTGCACGATA	ATGAGCACGCAGGGATTG	XM_067091896.1	108
scarb	CCTCACCACTGCCTTCTC	CCAACAATACCGTCACCTAC	XM_067132256.1	203
nos	CCCTTCAACGGGTGGTAC	AGGCTGGCTGGAGATTTT	XM_067116832.1	125
lec3	GCGACTCCTGTCGTCTGAAA	CCGTTTTGCCGTAGAGTCCT	XM_067106684.1	130
hemocyanin1	GAGAACCGTCTTCCTTGA	GCTCCTCATACCTCCTACA	XM_067113210.1	166
EF-1α	TGGACGTGTGGAGACTGGCATC	ATCGCCTGGAACAGCCTCAGTC	XM_067129063.1/	128

[1] alf2, anti-lipopolysaccharide factor 2; hsp70, heat shock protein 70; clec1, c-type lectin 1 ; lec3, lectin 3; hemocyanin1, hemocyanin subunit 1; scar, scavenger receptor; scarb, scavenger receptor b; serpin1, serine protease inhibitor 1; nos, nitric oxide synthase; propo, prophenoloxidase; sod, superoxide dismutase.

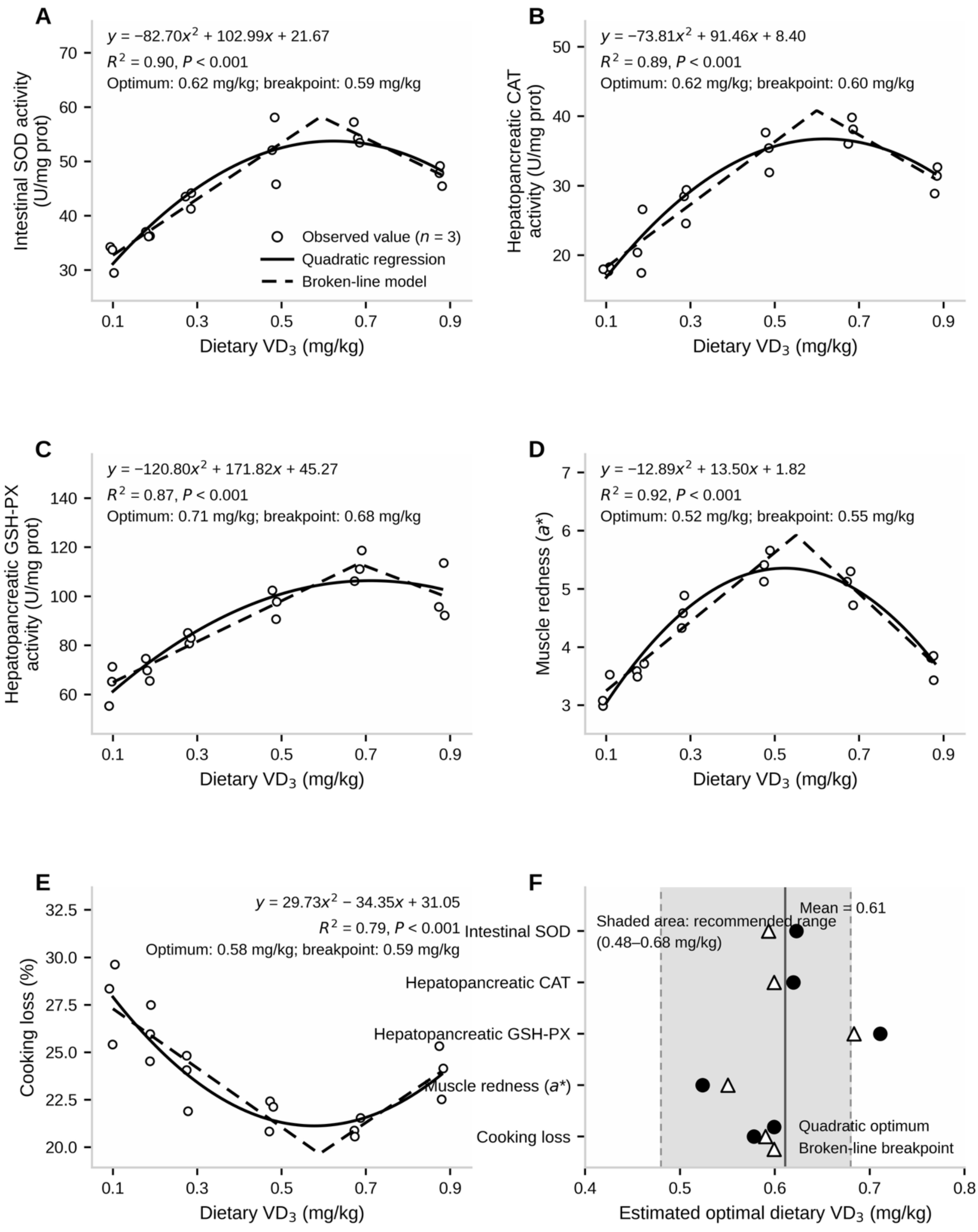

Supplementary Figure S1. One-way ANOVA and polynomial (quadratic) regression analysis of different muscle parameters.
